# Serum Neutrophil Gelatinase-Associated Lipocalin (NGAL) Could Provide Better Accuracy Than Creatinine in Predicting Acute Kidney Injury Development in Critically Ill Patients

**DOI:** 10.3390/jcm10225379

**Published:** 2021-11-18

**Authors:** Edison Jahaj, Alice G. Vassiliou, Maria Pratikaki, Parisis Gallos, Zafeiria Mastora, Ioanna Dimopoulou, Stylianos E. Orfanos, Philippos Orfanos, Pagona Lagiou, Anastasia Kotanidou

**Affiliations:** 1 First Department of Critical Care Medicine & Pulmonary Services, School of Medicine, National & Kapodistrian University of Athens, Evangelismos Hospital, 10676 Athens, Greece; Edison.jahaj@gmail.com (E.J.); alvass75@gmail.com (A.G.V.); zafimast@yahoo.gr (Z.M.); idimo@otenet.gr (I.D.); sorfanos@med.uoa.gr (S.E.O.); 2 Department of Biochemistry, Evangelismos Hospital, 10676 Athens, Greece; mpratikaki@yahoo.com; 3 Computational Biomedicine Laboratory, Department of Digital Systems, University of Piraeus, 18534 Piraeus, Greece; parisgallos@yahoo.com; 4 Department of Hygiene, Epidemiology and Medical Statistics, School of Medicine, National and Kapodistrian University of Athens, 11527 Athens, Greece; phorfanos@med.uoa.gr (P.O.); pdlagiou@med.uoa.gr (P.L.); 5 Department of Epidemiology, Harvard T.H. Chan School of Public Health, Boston, MA 02115, USA

**Keywords:** acute kidney injury, biomarkers, ICU, creatinine, prognosis, NGAL

## Abstract

Acute kidney injury (AKI) is one of the most common complications in critically ill patients. In recent years, studies have focused on exploring new biomarkers for the early diagnosis and prognosis of AKI. The aim of this study was to investigate serum prognostic biomarkers (neutrophil gelatinase-associated lipocalin, NGAL, and creatinine) of AKI in critically ill patients. The study included 266 critically ill, initially nonseptic, patients admitted to a multidisciplinary ICU. Serum levels of NGAL and creatinine were measured at ICU admission. ROC curves were generated to estimate the prognostic value of the biomarkers, while a logistic regression analysis was performed to assess their association with an increased AKI risk. Patients were divided in two groups based on the development (*n* = 98) or not (*n* = 168) of AKI during their ICU stay. Serum NGAL levels at ICU admission were significantly higher in those who subsequently developed AKI compared to those who did not (*p* < 0.0001). NGAL was shown to be more accurate in predicting AKI development than creatinine; furthermore, NGAL levels were associated with an increased risk of AKI development (1.005 (1.002–1.008), *p* < 0.0001). In the present study, we were able to demonstrate that increased serum NGAL levels at ICU admission might be predictive of AKI development during ICU hospitalization. Further studies are needed to support NGAL as a prognostic marker of acute kidney injury.

## 1. Introduction

The three most common organ dysfunctions in sepsis are cardiovascular (shock), renal (acute kidney injury, AKI), and respiratory, each associated with a very high morbidity and mortality [1]. AKI is considered a clinical syndrome and comprises an important complication of patients, ranging from 5% in patients hospitalized in wards to up to 30% in intensive care unit (ICU) patients [2]. AKI cannot be prognosticated early in the critically ill, as opposed to coronary patients whose early indices (e.g., troponin) give the possibility to recognize the underlying pathological condition. Functional genomics and proteomics in experimental AKI models have recognized genes and gene products that can be used as biomarkers. These include a serum panel (neutrophil gelatinase-associated lipocalin, NGAL; and cystatin C), and a urine panel (NGAL; interleukin-18; and kidney injury molecule-1, KIM-1) [3]. These biomarkers, however, should be validated in larger cohorts, from multiple clinical settings. In clinical practice, AKI diagnosis is based on the elevated values of serum creatinine, the decrease in hourly diuresis, and the elevated levels of urea in the patient’s blood [4]. Serum creatinine can be an insensitive indicator of kidney injury; furthermore, its use is also limited by the absence of baseline values in many patients. Although sepsis is the most common contributor to AKI, it has been demonstrated that sepsis frequently develops after AKI in critically ill patients, suggesting that AKI may increase the risk of sepsis [5]. Both sepsis and AKI are clinical diagnoses, and it is usually difficult to define the precise time when these syndromes start to develop.

The aim of the present study was to investigate the prognostic value of experimental and clinical AKI biomarkers at ICU admission (NGAL and creatinine) in initially nonseptic critically ill patients and, furthermore, to examine the effect of NGAL levels on the risk of developing AKI.

## 2. Materials and Methods

This prospective, observational study was approved by the Hospital’s Ethics Committee (study approval number 48–3/03/2017), and all procedures carried out were according to the Helsinki Declaration. All patients’ next-of-kin provided us with their informed written consent.

The study population consisted of 266 critically ill patients, hospitalized from 2017 to 2020 in the intensive care unit (ICU) of “Evangelismos” Hospital. The inclusion criteria included adult patients (>18 years old) and an ICU stay longer than 5 days. The exclusion criteria included the presence of acute kidney failure within the first 24 h post ICU admission, sepsis within the first 24 h postadmission, BMI > 35 kg/m^2^, pregnancy, brain death, patients with final stage cancer or chronic kidney failure, re-admission or transfer from another ICU, and infectious diseases.

Patients were grouped in two categories: those who developed AKI during their ICU stay, and those who did not, who were used as a comparison group. AKI was defined using the criteria of serum creatinine or glomerular filtration rate (GFR) and urine output, according to RIFLE (Risk, Injury, Failure, Loss, End-stage renal disease) [6]. The patients were monitored daily for the development of AKI.

Three milliliters of venous blood was collected within the first 24 h post ICU admission. The blood was collected in EDTA tubes. NGAL was measured using the immunofluorescent Triage^®^ NGAL Test (Biosite Inc., San Diego, CA, USA). The assay has a detection range of 60–1300 ng/mL.

Data are given as mean ± SD, or median and interquartile range (IQR). The Student’s *t* test, the nonparametric Mann–Whitney test, or the Fisher’s exact test were used to compare the two groups, as appropriate. Initially, a univariate logistic regression model was used to evaluate the association of NGAL with the development of AKI. Afterwards, a multivariate logistic regression model was performed to evaluate the association of NGAL with AKI development in the presence of potential confounders, namely, age, sex, SOFA score, and duration of ICU stay. A receiver operating characteristic (ROC) curve was used for the detection of the prognostic value of NGAL. *p*-values < 0.05 were considered statistically significant. All analyses were performed with SPSS v24 (IBM Statistics, New York, NY, USA).

## 3. Results

**Figure 1:**
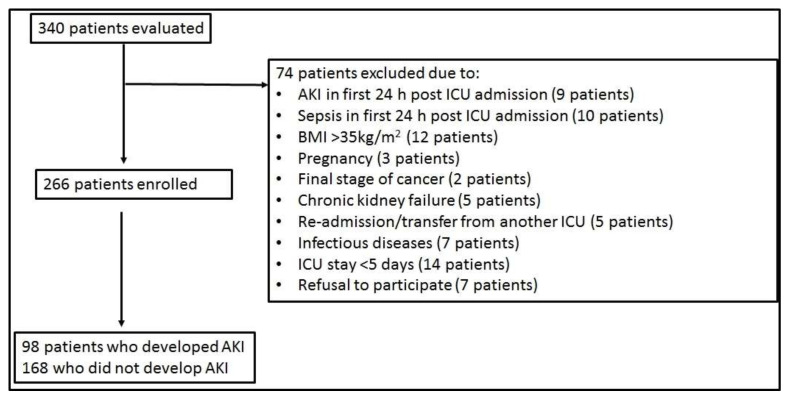
Flowchart of the study’s enrollment. Definition of abbreviations: AKI = Acute kidney injury; BMI = Body mass index; ICU = Intensive care unit.

Out of 340 patients screened, eventually 266 initially nonseptic patients were enrolled in the study (see Figure 1). The majority of patients were male (75%), and the mean age was 47 years. Sixty-four percent (64%) were trauma patients. Patients were subsequently grouped into those who developed AKI (*n* = 98) or not (*n* = 168) during their ICU stay. The two groups differed in terms of age, disease severity (acute physiology and chronic health evaluation, APACHE II, and sequential organ failure assessment, SOFA, scores), and duration of ICU stay (Table 1). Sepsis developed after 6 (4–9) days in the whole ICU cohort. The AKI group consisted primarily (97%) of patients with sepsis-induced AKI (S-AKI). In three patients, AKI developed prior to sepsis. In the non-AKI group, 45% of the patients developed sepsis during their ICU stay without developing AKI. Finally, in the AKI group, 29 patients (29.6%) required renal replacement therapy (RRT); ICU admission NGAL levels could not differentiate patients who eventually required RRT or not (140 (65–221) ng/mL vs. 123 (60–325) ng/mL; *p* > 0.05).

**Figure 2:**
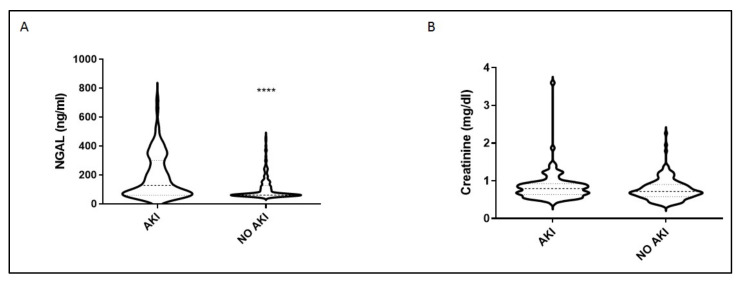
Intensive care unit (ICU) admission levels of NGAL and creatinine. (**A**) NGAL and (**B**) creatinine were measured at ICU admission (within 24 h) in 98 critically ill patients who eventually developed AKI in the ICU and in 168 patients who did not. Two-group comparisons were performed with the non-parametric Mann–Whitney test, **** *p* < 0.0001. Data are presented as violin plots, indicating the median value, and 25th and 75th centiles. Definition of abbreviations: AKI = Acute kidney injury; NGAL = Neutrophil gelatinase-associated lipocalin.

**Figure 3:**
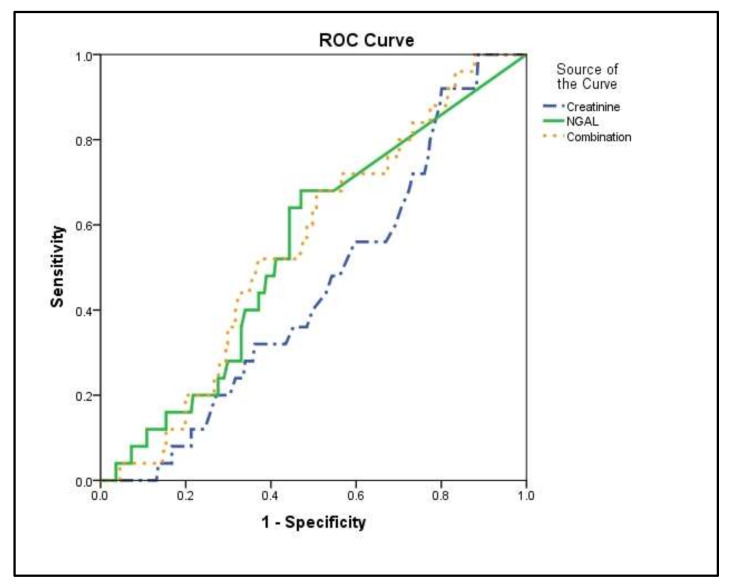
ICU admission biomarker levels and AKI development. Receiver operating characteristic (ROC) curve analysis. ROC curves were generated to determine the prognostic accuracy of either NGAL (solid line), creatinine (dash–dotted line), or their combination (dotted line), as measured at ICU admission (within 24 h). Definition of abbreviations: AKI = Acute kidney injury; NGAL = Neutrophil gelatinase-associated lipocalin.

Most importantly, serum NGAL levels at ICU admission were much higher in the AKI group compared with the non-AKI group (*p* < 0.0001; Figure 2a). The ICU admission serum creatinine values did not differ in the two groups (*p* = 0.1; Figure 2b). The prognostic value of NGAL was evaluated by generating ROC curves. In these, NGAL showed a higher accuracy in prognosticating the development of AKI compared to creatinine (Table 2 and Figure 3). The linear combination of NGAL with creatinine did not improve prognostic accuracy (*p* > 0.05; Table 2 and Figure 3).

In order to further investigate the association of NGAL levels with the possibility of developing AKI, we performed univariate and multivariate logistic regression analyses (Table 3). As can be seen, the univariate analysis indicated that NGAL levels were associated with a higher probability of developing AKI (OR = 1.006, 95%; CI = 1.004–1.009; *p* < 0.0001). The multivariate model included other possible independent confounders, such as age, sex, SOFA score, and ICU stay duration. The multivariate analysis showed that NGAL levels could be assumed as independent predictors of AKI development (1.005 (1.002–1.008), *p* < 0.0001) in the presence of the duration of ICU stay.

It is worth noticing that women tended to have a double risk of developing AKI compared to men (without reaching statistical significance, probably due to the limited sample size; Table 3). Additionally, the univariate analysis indicated that SOFA score is associated with a higher probability of developing AKI; however, in the multivariate analysis, it was not considered an independent factor, probably due to the effect of other confounders.

## 4. Discussion

In the present prospective study, we investigated the value of ICU admission serum NGAL levels in the prognosis of acute kidney injury in initially nonseptic critically ill patients. Our results support the notion that serum NGAL might be a promising biomarker for AKI prognosis in critically ill patients.

NGAL was originally isolated from human neutrophils as a 25 KDa protein linked to matrix metalloproteinase-9 [7]. NGAL has been extensively investigated in various AKI phenotypes. In an earlier report, the diagnostic accuracy of plasma NGAL in predicting AKI in emergency department (ED) patients with suspected sepsis was demonstrated [8]. In critically ill patients, various reports have demonstrated that serum NGAL ICU admission levels could be used as an early biomarker of AKI in adult critically ill patients [9,10,11,12,13]. Specifically, NGAL measured at ICU admission could predict AKI development similarly to serum creatinine-derived eGFR, and furthermore, NGAL added significant accuracy to the prediction in combination with eGFR alone or with other clinical parameters [9]. In another study, in critically ill patients without pre-existing kidney disease, ICU admission plasma NGAL could also predict AKI occurrence up to 72 h post ICU admission with a fair performance [14]. In a surgical ICU, a pilot study demonstrated that a panel including serum NGAL, urinary NGAL, and SOFA scores could allow early diagnosis with high sensitivity and specificity, prognosis for septic AKI, and in-hospital mortality [15]. Some studies have found that plasma NGAL failed to discriminate adult and children patients with AKI from those with non-AKI in a sepsis setting [16]. Studies have also shown limited sensitivity and specificity of NGAL in distinguishing AKI from chronic kidney disease (CKD) [16]. The results of these studies prompted the researchers to suggest that NGAL levels should be used cautiously as AKI predictors in general ICU patients.

Since plasma NGAL may also increase in systemic infection and inflammation without evidence for AKI, we measured ICU admission NGAL levels in initially nonseptic critically ill patients. In our cohort, AKI was primarily due to sepsis development during ICU stay. Sepsis has a defined pathophysiology, which makes S-AKI a distinct syndrome from any other phenotype of AKI [16]. S-AKI is usually defined as AKI development in the presence of sepsis without other significant contributing factors explaining AKI. Hence, the definition of S-AKI requires the diagnosis of sepsis and subsequent occurrence of AKI [17]. The current definition for AKI based on serum creatinine and urine output is limited by the delayed identification of such patients.

In our study, an increase in NGAL levels by 10 ng/dL caused an increased risk of AKI of about 5%, controlling for potential confounders. Moreover, length of stay in the ICU was another important factor that contributed to AKI. More specifically, our results showed that one more day in the ICU caused an 11% increase in the risk of AKI. Hence, our results agree with the studies that render a role to NGAL as being able to predict AKI in critically ill patients several hours before clinical diagnosis. Furthermore, we demonstrated that the increased NGAL ICU admission levels in the patients who will develop AKI have a better predictive value than creatinine levels, while the addition of ICU admission creatinine levels to the ROC model for predicting AKI development showed no significant additive value.

The limitations of our study must be stated. This was a single-center study, with a moderate sample size. NGAL was measured within 24 h post ICU admission; it has been shown that a measurement within 2–4 h can capture earlier changes that occur with kidney injury. However, studies have shown that there are no differences in NGAL performance when measured earlier within the ED or later in the ICU. Finally, we recorded the day that sepsis developed in our patients; however, due to delayed identification of AKI, we did not record the precise day that AKI developed. Hence, we could not perform survival curve (Kaplan–Meier or Cox) comparisons for AKI prediction based on ICU admission NGAL levels.

## 5. Conclusions

Serum creatinine, a commonly used marker for the diagnosis of AKI, due to delayed increase is unable to accurately estimate the timing of injury. Therefore, there is a need for a reliable AKI biomarker, which should be specific and sensitive, be capable of capturing early change following kidney injury, and be easy to measure. The data presented herein support that serum NGAL could be a useful early prognostic biomarker for AKI development in a general, heterogeneous, adult ICU patient population.

## Figures and Tables

**Table 1:** Demographics, clinical and laboratory characteristics of the two patient groups at ICU admission, and important outcomes.

Parameters	AKI	Non-AKI	*p*-Value
Number of patients, (*n*)	98	168	
ICU admission			
Age (years), (median, IQR)	51 (34–63)	45 (29–59)	0.066
Sex, *n* (%)			0.400
Male	71 (72)	128 (76)	
Female	27 (28)	40 (24)	
Diagnosis, *n* (%)			0.900
Medical	20 (20)	20 (12)	
Surgical	21 (22)	35 (21)	
Trauma	57 (58)	113 (67)	
APACHE II score, (mean ± SD)	16 ± 6	14 ± 5	0.003 *
SOFA score, (median, IQR)	7 (6–9)	6 (5–8)	0.047 *
PaO_2_/FiO_2_ (mmHg), (mean ± SD)	322.3 ± 132.3	343.1 ± 141.5	0.300
NGAL (ng/mL), (median, IQR)	127 (60–300)	60 (60–127)	<0.0001 *
Creatinine (mg/dL), (median, IQR)	0.79 (0.64–0.92)	0.72 (0.58–0.90)	0.200
ICU course			
Sepsis during ICU stay, *n* (%)	95 (96.9%)	75 (44.6%)	<0.0001 *
Sepsis day, (median, IQR)	6 (4–8)	6 (3–10)	0.610
Renal replacement therapy, *n* (%)	29 (29.6%)	N/A	
LoS in the ICU (days), (median, IQR)	32 (25–41)	11 (7–20)	<0.0001 *
Mortality, *n* (%)	13 (13%)	16 (10%)	0.400

* *p* < 0.05. Data are presented as number of patients (N) and percentages (%) or as mean ± SD for the normally distributed variables, or as median and interquartile range for skewed distributed variables. The Fisher’s exact test was used for the categorical variables, while the Student’s *t* test was performed for normally distributed variables, and the Mann–Whitney nonparametric test was performed for skewed variables. APACHE II, SOFA score, PaO_2_/FiO_2_, creatinine, and NGAL were estimated within 24 h from ICU admission. Sepsis developed subsequently in 170 initially nonseptic patients during ICU stay, while the sepsis day denotes the day that sepsis developed following ICU admission. Definitions of abbreviations: AKI = Acute kidney injury; APACHE = Acute physiology and chronic health evaluation; ICU = Intensive care unit; LoS = Length of stay; NGAL = Neutrophil gelatinase-associated lipocalin; SOFA = Sequential organ failure assessment; N/A = Not applicable.

**Table 2:** Prognostic values of AKI biomarkers.

Parameters	Cut-Off Value	AUC	95% CI	*p*-Value	Specificity%	Sensitivity%
NGAL (ng/mL)	90.5	0.67	0.60–0.74	<0.0001	61.78	59.14
Creatinine (mg/dL)	0.77	0.56	0.49–0.63	0.100	57.86	53.61
NGAL + Creatinine		0.67	0.59–0.74	<0.0001	66.67	59.14

**Table 3:** Odds ratios and 95% confidence intervals for the possible prognostic factors of AKI development.

Variables	Univariate Model			Multivariate Model		
	OR	95% CI	*p*	OR	95% CI	*p*
Age (years)	1.011	0.998–1.025	0.10	1.007	0.989–1.026	0.434
Sex						
Male	Ref. value			Ref. value		
Female	1.280	0.728–2.250	0.40	2.235	0.999–4.998	0.050
SOFA score	1.116	1.003–1.242	0.04 *	0.878	0.750–1.028	0.106
NGAL (ng/mL)	1.006	1.004–1.009	<0.0001 *	1.005	1.002–1.008	0.0001 *
LoS in the ICU (days)	1.116	1.085–1.148	<0.0001 *	1.112	1.078–1.148	<0.0001 *

* *p* < 0.05. A univariate logistic regression model was used to evaluate the association of NGAL and other factors with the development of AKI. Afterwards, a multivariate logistic regression model was used to evaluate the association of NGAL with AKI development in the presence of potential confounders, namely, age, sex, SOFA score, and length of stay in the ICU. Definitions of abbreviations: AKI = Acute kidney injury; ICU = Intensive care unit; LoS = Length of stay; NGAL = Neutrophil gelatinase-associated lipocalin; SOFA = Sequential organ failure assessment.

## Data Availability

Data are contained within the article.
